# Prevalence, Serotypes, Antimicrobial Resistance and Biofilm-Forming Ability of *Listeria monocytogenes* Isolated from Bulk-Tank Bovine Milk in Northern Greece

**DOI:** 10.3390/pathogens12060837

**Published:** 2023-06-17

**Authors:** Apostolos S. Angelidis, Afroditi S. Grammenou, Charalampos Kotzamanidis, Nektarios D. Giadinis, Antonios G. Zdragas, Daniel Sergelidis

**Affiliations:** 1 Laboratory of Safety and Quality of Milk and Dairy Products, School of Veterinary Medicine, Aristotle University of Thessaloniki, 54124 Thessaloniki, Greece; asangel@vet.auth.gr; 2 Laboratory of Hygiene of Foods of Animal Origin-Veterinary Public Health, School of Veterinary Medicine, Aristotle University of Thessaloniki, 54124 Thessaloniki, Greece; afrogramm@yahoo.gr; 3 Hellenic Agricultural Organization-DIMITRA, Veterinary Research Institute of Thessaloniki, 57001 Thermi, Greece; kotzam@vri.gr (C.K.); zdragas@vri.gr (A.G.Z.); 4 Clinic of Farm Animals, School of Veterinary Medicine, Faculty of Health Sciences, Aristotle University of Thessaloniki, 54627 Thessaloniki, Greece; ngiadini@vet.auth.gr

**Keywords:** *Listeria*, raw milk, biofilm, prevalence, antimicrobial resistance, Greece

## Abstract

The prevalence of *Listeria monocytogenes* in bovine bulk-tank milk (BTM) in Greece has not been previously investigated. The aim of the study was to estimate the prevalence of *L. monocytogenes* in bovine BTM in Greece and to characterize the isolates in terms of carriage of genes encoding for pathogenic determinants, assess the isolates’ biofilm-forming ability and determine their susceptibility against 12 antimicrobials. Samples (n = 138) of bovine BTM were obtained from farms located throughout Northern Greece and were analyzed qualitatively and quantitatively for *L. monocytogenes*. Five samples (3.6%) tested positive for *L. monocytogenes*. The pathogen’s populations in these positive samples were below 5 CFU/mL. Most isolates belonged to the molecular serogroup “1/2a, 3a”. All isolates carried the virulence genes *inlA*, *inlC*, *inlJ*, *iap*, *plcA* and *hlyA*, but *actA* was detected in only three isolates. The isolates displayed weak to moderate biofilm-forming ability and distinct antimicrobial resistance profiles. All isolates were characterized as multidrug resistant, with resistance to penicillin and clindamycin being a common feature. Considering that *L. monocytogenes* constitutes a serious public health threat, the key findings of the study, related to the carriage of virulence genes and multidrug resistance, highlight the importance of continued monitoring of the pathogen in farm animals.

## 1. Introduction

Out of the 28 different species in the genus *Listeria* that have been identified to date [1,2] only *Listeria monocytogenes* is considered to be pathogenic to humans. *L. monocytogenes* is the causative agent of listeriosis, a serious food-borne disease in humans and domestic ruminants [3]. In Greece, the notification rate of listeriosis in humans is low (1.2 cases per 1,000,000 population), probably due to underreporting of the surveillance system. According to data from the National Public Health Organization (NPHO) [4], for the period 2004–2021, a total of 231 cases of listeriosis were reported, with a mean annual number of cases equal to 12.7 ± 8.1. However, according to the latest available annual report of the European Food Safety Authority (EFSA) and the European Centre for Disease Prevention and Control (ECDC), 27 European Member States reported 2183 confirmed cases of invasive listeriosis in humans in 2021, with 923 hospitalizations and 196 deaths, and an overall high (13.7%) European Union (EU) case-fatality rate. Thus, listeriosis still constitutes one of the most serious food-borne human diseases under EU surveillance [5].

Unlike the case of ready-to-eat (RTE) foods where microbiological food-safety criteria, established by Regulation (EC) 2073/2005 [6], are used to assess the acceptability of manufacturing processes and foodstuffs, there exist neither harmonized surveillance of *L. monocytogenes* and listeriosis in feed and animals in the EU, respectively, nor harmonized surveillance of antimicrobial resistance in *L. monocytogenes* [7]. Raw milk can become directly contaminated with *L. monocytogenes* from lactating animals with mastitis due to *L. monocytogenes* [8]. Affected cows can shed high populations of the pathogen in their milk [9], and they can be persistently infected, leading to prolonged contamination of the bulk-tank milk (BTM) [10]. Alternatively, under unhygienic animal-milking practices, contamination of raw milk can occur indirectly from animal feeds, feces, bedding or contaminated udder surface [11], and the risk of contamination of milk with *L. monocytogenes* on dairy farms has been shown to increase with feeding poor-quality silage and employing insufficient animal housing and milking hygiene [12].

In 2021 in the EU, 1.4% out of 138 tested batches and 20% out of 10 single units of raw milk intended for direct human consumption tested positive for *L. monocytogenes* [5]. Pasteurization of raw milk can effectively eliminate [13] the low levels of *L. monocytogenes* that may occasionally be present [14]. Nevertheless, application of improper pasteurization schemes or rare instances of post-pasteurization contamination (e.g., during the filling and packaging of pasteurized milk in the manufacturing plants) can lead to the presence of viable *L. monocytogenes* in pasteurized milk. For instance, in 2015, 6.0% of the batches of pasteurized bovine milk sampled at the processing-plant level in Poland tested positive for *L. monocytogenes* [15]. Due to its elaborate cold-adaptation mechanisms, *L. monocytogenes* can readily grow in pasteurized milk stored under refrigeration [16].

To the best of our knowledge, the prevalence of *L. monocytogenes* in the bulk tanks of bovine farms in Greece has not been previously investigated. Therefore, the aim of the study was to estimate the prevalence of *L. monocytogenes* in bovine BTM in Greece and to characterize the isolates in terms of carriage of genes encoding for pathogenic determinants, assess the isolates’ biofilm-forming ability and determine their susceptibility against selected antimicrobials, including those typically used for the treatment of listeriosis in humans.

## 2. Materials and Methods

### 2.1. Sampling

Between June and July of 2019, samples of raw milk were obtained from the bulk tanks of 138 bovine farms located throughout Northern Greece. Prior to milk sampling, the bulk-tank contents were mechanically agitated for at least 5 min. Milk samples (100 mL) were then placed in sterile plastic containers with lids and transferred to the laboratory under refrigeration (in insulated containers containing ice packs, at *ca*. 2–4 °C) within 2–6 h after sampling.

### 2.2. Microbiological Analyses

Samples were immediately analyzed upon arrival at the laboratory. A 25-mL portion of each sample was analyzed qualitatively for the presence of *L. monocytogenes* according to ISO 11290-1 [17]. Half-Fraser broth and Fraser broth (Biolife Ialiana S. r. l., Milano, Italy) and Agar *Listeria* according to Ottaviani and Agosti (ALOA; LabM, Lancashire, UK) were used as the primary enrichment broth, secondary enrichment broth and selective solid medium, respectively. At the same time, 0.2 mL of undiluted milk from each BTM sample were spread-plated onto the surface of ALOA agar plates and incubated at 37 °C for up to five days for the enumeration of *L. monocytogenes* (quantitative analysis).

Presumptive *L. monocytogenes* colonies on ALOA (bluish-green colonies surrounded by an opaque halo) were sub-cultured (at 37 °C for 24 h) onto Tryptone Soya Yeast Extract Agar (TSYEA; Biolife) and subjected to the mandatory biochemical confirmation tests (microscopic aspect, β-hemolysis test, L-Rhamnose and D-Xylose fermentation tests) and to two of the optional biochemical confirmation tests (catalase test and motility assessment at 25 °C) specified by ISO 11290-1. Columbia agar with sheep blood plates (Oxoid, Deutschland, GmbH) were used for assessing β-hemolysis. Phenol-red broth base (Millipore, Merck KGaA, Darmstadt, Germany) and L-Rhamnose and D-Xylose discs (Rosco, Albertslund, Denmark) were used for carbohydrate fermentation tests.

One confirmed *L. monocytogenes* isolate from each *L. monocytogenes*-positive BTM sample was also grown (at 37 °C for 24 h) in Tryptone Soya Yeast Extract Broth (TSYEB; Biolife) and stored at −80 °C in 20% glycerol for further characterizations. For the molecular characterization of the isolates, DNA for PCR analyses was extracted from overnight TSYEB cultures using a DNA purification protocol (Pure Link Genomic DNA kit, Invitrogen, Carlsbad, CA, USA).

### 2.3. Molecular Serotyping of the L. monocytogenes Isolates

*L. monocytogenes* isolates were molecularly serotyped using the multiplex-PCR protocol (primer sets, reagent concentrations and amplification conditions) of Doumith et al. [18] which targets the *lmo0737*, *lmo1118*, ORF2819 and ORF2110 serotype-specific genes. The *L. monocytogenes* strains previously serotyped by Kotzamanidis et al. [19] were used as positive controls.

### 2.4. Detection of Selected Virulence Genes in the L. monocytogenes Isolates

The presence of seven virulence-associated genes were investigated by multiplex-PCRs using two sets of primers. The *inlA*, *inlC* and *inlJ* genes were detected using the primers and the PCR conditions described by Liu et al. [20], while for the detection of *plcA*, *actA*, *hlyA* and *iap* genes, the PCR protocol described by Rawool et al. [21] was used. Genomic DNA from *L. monocytogenes* strains previously characterized by Kotzamanidis et al. [19] were used as positive controls.

The PCR reactions for molecular serotyping and detection of virulence genes were performed in a Thermal Cycler (model MJ96, Applied Biosystems, Thermo Fisher Scientific Inc., Waltham, MA, USA). The DNA amplification products were analyzed using agarose (1.0%) gel electrophoresis, stained with ethidium bromide (0.5 mg/mL) and visualized in a gel documentation system (Bio-Rad Laboratories Inc., Hercules, CA, USA).

### 2.5. Antimicrobial Susceptibility Testing of the L. monocytogenes Isolates

The antimicrobial susceptibility testing of the isolates was performed according to the recommendations of the Clinical and Laboratory Standards Institute (CLSI) [22] using the disc-diffusion method on Mueller–Hinton agar plates supplemented with defibrinated sheep blood (Merck). The breakpoints of *Staphylococcus* spp. resistance were used as previously described [23] because, with the exception of ampicillin, penicillin and trimethoprim–sulfamethoxazole for which susceptibility breakpoints are provided, no other interpretative criteria are available for *Listeria* in the CLSI guidelines. The inoculum was standardized using spectrophotometry (Spectronic 20, Bausch & Lomb, Rochester, New York, NY, USA), and the plates were inoculated with a concentration of *ca.* 8 log_10_ CFU per mL using sterile cotton swabs. The inoculated plates were incubated at 35 °C for 24 h. The *L. monocytogenes* isolates were tested against a panel of 12 antimicrobials (Oxoid Ltd., Basingstoke, UK) that are commonly used in human and veterinary medicine. The antimicrobials and their corresponding concentrations were amoxicillin/clavulanic acid (A/C, 20/10 μg), ampicillin (AMP, 10 μg), ciprofloxacin (CIP, 5 μg), chloramphenicol (C, 30 μg), clindamycin (CLI, 2 μg), erythromycin (ERY, 15 μg), gentamicin (G, 10 μg), oxacillin (OX, 1 μg), penicillin (P, 10 IU), sulphamethoxazole/trimethoprim (SXT, 1.25/23.75 μg), tetracycline (TET, 30 μg) and vancomycin (VAN, 30 μg). *S. aureus* ATTC 25923 was used as the positive control strain. Multidrug resistance (MDR) was defined as non-susceptibility of an isolate to at least one agent in three or more antimicrobial categories, according to the criteria proposed by Magiorakos et al. [24].

### 2.6. Assessment of the Biofilm-Forming Ability of the L. monocytogenes Isolates

The ability of the *L. monocytogenes* isolates to form biofilms was tested using the semi-quantitative adherence protocol published by Wang et al. [25]. In brief, the protocol relies on optical density (OD) readings (at 570 nm) of adherent biofilms that have been stained with 0.3% (*w*/*v*) crystal violet solution in individual wells of polystyrene microtiter plates. The average OD value of plain broth medium (negative control) was used to define the cut-off OD (OD_c_) value. Depending on the assay’s results (resulting OD readings of each strain), the *L. monocytogenes* isolates were classified according to Borges et al. [26] either as no biofilm producers (OD < OD_c_), weak biofilm producers (when OD_c_ < OD ≤ 2 × OD_c_), moderate biofilm producers (when 2 × OD_c_ < OD ≤ 4 × OD_c_) or strong biofilm producers (when 4 × OD_c_ < OD).

### 2.7. Statistical Analysis

The exact probability method of Minitab (version 17, Minitab Inc., State College, PA, USA) was used to calculate the 95% confidence interval (CI) for the estimated proportion of *L. monocytogenes*-positive BTM samples.

## 3. Results and Discussion

### 3.1. Isolation Frequency and Populations of L. monocytogenes in Bovine Bulk-Tank Milk

Five out of the 138 samples (3.6%; 95% CI = 1.2–8.3%) of bovine BTM tested positive for the presence of *L. monocytogenes*. Based on the results of the quantitative analysis, the populations of *L. monocytogenes* in the *L. monocytogenes*-positive samples were below the limit of enumeration of our assay (<5 CFU/mL).

The isolation frequency of *L. monocytogenes* from bovine BTM in our survey is comparable to most of the corresponding estimates reported in Europe [27,28,29,30,31] based on literature published in recent (2010–2023) years (Table 1). A much higher prevalence estimate (18.1%) was reported for raw milk in Estonia, but the source and animal-origin of the raw milk analyzed was not specified [32]; in the same study, enumeration analyses in 230 raw milk samples revealed that the populations of *L. monocytogenes* were <10 CFU/g in almost all (99.6%) of the samples. Regardless of the source of contamination of raw milk with *L. monocytogenes*, the contaminant populations are dispersed in a large volume of milk in the bulk tank, and consequently, the initial cell density of the pathogen in BTM is typically low, i.e., from less than 1 CFU/mL up to a few dozen CFU/mL [31,33,34,35,36,37]. This was also the case in our study where none of the five *L. monocytogenes*-positive BTM samples nor any of the other 133 samples (that tested negative with the detection protocol) contained enumerable populations of *L. monocytogenes*.

### 3.2. PCR-Serogrouping of the L. monocytogenes Isolates

Fourteen different serotypes are currently recognized within *L. monocytogenes* [40]. The molecular method used for serotyping the isolates [18] is based on the detection of four *L. monocytogenes* marker genes (*lmo1118*, *lmo0737*, ORF2110 and ORF2819) via multiplex-PCR in order to classify the four major *L. monocytogenes* serovars (1/2a, 1/2b, 1/2c, 4b) into four distinct “PCR-serogroups”, i.e., distinct PCR amplification profiles [41]. At the same time, the amplification of the *prs* gene (in the same multiplex-PCR reaction) serves as a confirmation of the isolates to the genus level (*Listeria* spp.). In our study, the five *L. monocytogenes* isolates were classified into three different serogroups: three isolates belonged to serogroup IIa, one isolate belonged to serogroup IIc and the last isolate to the serogroup IVb (Table 2). The *Listeria* spp.-specific marker *prs* was detected in all *L. monocytogenes* isolates.

In Greece, a limited number of studies have provided characterization data (serotypic and genotypic diversity, carriage of virulence genes, antibiotic resistance or biofilm-forming ability) of *L. monocytogenes* strains isolated from different sources such as meat, meat products, meat-processing environment and vegetables [42,43], soft whey-cheese and related food-processing surfaces [44], veterinary samples and raw ovine milk [19] or human clinical cases [45]. However, to our knowledge, data on *L. monocytogenes* isolates from bovine milk in Greece are not available.

Although the number of positive samples and the concomitant characterized isolates in our study is too small to make any generalizations, it appears that the serogrouping classification of our isolates is in line with the findings and trends of recently published pertinent studies (*L. monocytogenes* isolates from bovine BTM) from other countries where the majority of isolates were classified to the IIa (“1/2a, 3a”) molecular serogroup [46,47,48]. However, only one of the five *L. monocytogenes* isolates from BTM in Rajasthan, India, was classified to serogroup “1/2a, 3a” where most (three out of five) of the isolates belonged to the “4b, 4d, 4e” serogroup [49]. Kramarenko et al. [32] used specific antisera for serotyping 46 *L. monocytogenes* isolates obtained from raw milk in Estonia between 2008 and 2010. The authors reported that the majority of the isolates (37) were of serotype 1/2a, and the remaining nine isolates belonged to serotypes 1/2b, 4b, 1/2c and 4d.

The vast majority of *L. monocytogenes* strains that have been isolated from human listeriosis cases (both outbreak and sporadic) belong to serotypes 1/2a, 1/2b, and 4b [50]. More recently, a review by Lopez-Valladares et al. [51] noted a shift from serotype 4b to serotype 1/2a in human listeriosis cases in Europe and Northern America, whereas according to a recent literature review [52], the most frequent serotypes identified in 66 listeriosis outbreaks were 4b (62%) and 1/2a (29%).

### 3.3. Carriage of Genes Encoding for Virulence Factors in the L. monocytogenes Isolates

Although all *L. monocytogenes* strains are considered as (equally) pathogenic from a regulatory authority viewpoint, it has been well-established in the scientific literature that *L. monocytogenes* strains can differ considerably with respect to their virulence potential [53,54]. In the present study, all the virulence genes assayed for (*iap*, *inlA*, *inlC*, *inlJ*, *plcA*, *hlyA* and *actA*) were detected in all the *L. monocytogenes* isolates, except for two strains that tested negative for *actA* (Table 2). In Greece, the same set of seven virulence genes was detected in all of the 57 *L. monocytogenes* strains isolated from small-ruminant encephalitis cases and raw ovine milk in Greece [19] and in all (12) strains isolated from raw bovine meat, meat-processing surfaces and a food-handler, with the exception of four strains that had been isolated from the equipment and the meat-processing environment, which lacked *actA* [43]. The *actA* gene is a genetically diverse gene of the *L. monocytogenes* pathogenicity island I (LIPI-1) [55]. Poimenidou et al. [56] investigated the nucleotide sequence diversity of the six virulence genes encoded by LIPI-1 in *L. monocytogenes* isolates from different geographical locations, serotype and isolation source and reported that *actA* was the most diverse gene displaying the highest number of alleles among the genes tested. It may be that the two *actA*-negative isolates in our study harbored mutations or deletions in the *actA*-gene sequence, but further analyses would be required to check this hypothesis.

Several investigators have previously reported the carriage of these virulence determinants by *L. monocytogenes* isolates originating from raw bovine milk. Therefore, all seven virulence genes were detected in all *L. monocytogenes* isolates (10) from raw cow’s milk in Ghana [57]. With the exception of *iap* (which was not screened for), these virulence genes were detected in two isolates recovered from raw milk samples in the Eastern Cape Province of South Africa [58]. The internalin genes (*inlA*, *inlC*, *inlJ*) were also detected in all (13) *L. monocytogenes* isolates from raw cow’s milk in Iran [46] and in about half of the isolates from raw milk (22) and feces (6) in Jordan [59]; *inlA* and *inlC* (but not *inlJ*) were detected in all (4) bovine BTM isolates in India [49]; *hlyA* and *actA* were detected in all (6) *L. monocytogenes* isolates from bovine BTM in Kosovo [30].

The carriage of three internalin genes as well as of genes encoded by the LIPI-1 of *L. monocytogenes* in our isolates highlights their potential virulence capacity and threat to public health.

### 3.4. Biofilm-Forming Ability of the L. monocytogenes Isolates

In our survey, two of the *L. monocytogenes* isolates displayed moderate biofilm-forming ability, and the remaining three displayed weak biofilm-forming ability (Table 2). The low number of *L. monocytogenes* isolates in our study did not permit to assess any correlation between biofilm-forming ability and serotype. However, a study dedicated to biofilm-forming abilities of *L. monocytogenes* serotypes isolated from different (clinical and food) sources failed to detect any correlation between serotype and biofilm-forming ability [60]. Recently, Di Ciccio et al. [61] assessed the biofilm-forming ability of 57 *L. monocytogenes* isolates originating from food and food-processing environments in Italy and reported that although all isolates produced biofilms, most the strains were classified as weak or moderate biofilm-producers and noted that the percentage of isolates from meat-products that were deemed as either moderate or strong biofilm-producers was higher than that from dairy products. Biofilm formation by food-borne pathogenic microorganisms is a highly complex phenomenon that depends on many intrinsic and extrinsic factors [62]. Formation of biofilms and persistence of *L. monocytogenes* in the primary production or food-processing environment is a potential cause of repeated contamination of foods. For instance, in the dairy farm environment, the residence of *L. monocytogenes* in microbial biofilm matrices on the inner surfaces of the milking equipment may result in release of *L. monocytogenes* cells into the BTM [63].

### 3.5. Antimicrobial Susceptibility of the L. monocytogenes Isolates

The timely administration of an efficient antimicrobial treatment is necessary to prevent complications or death in human listeriosis patients [64]. Penicillin or an aminopenicillin (ampicillin or amoxicillin) combined with an aminoglycoside (gentamicin) are considered to be the first-choice treatment for listeriosis [65]. Alternative treatment choices, such as erythromycin, trimethoprim–sulfonamide (cotrimoxazole) or vancomycin, may be considered in case of penicillin allergy or depending on the age group or the clinical manifestations of listeriosis [3,52].

All the *L. monocytogenes* isolates in our study displayed resistance against some of the tested antimicrobials (from three up to six), with each isolate presenting a different antimicrobial resistance profile (ARP). Resistance towards P and CLI was noted in all isolates, followed by resistance to A/C and ERY (3 isolates), TET (2 isolates) and AMP, CIP and C (1 isolate). None of the isolates displayed resistance towards G, OX, SXT or VAN (Table 2). Based on their ARPs, all the isolates were characterized as MDR, displaying resistance to three or more (up to five) classes of antimicrobials.

With respect to the antimicrobial agents used in our study, the assessment of the antimicrobial susceptibility of *L. monocytogenes* strains that have been isolated from raw milk (bovine, caprine or ovine) and dairy products in recent years has revealed resistance frequencies ranging from zero up to 80% for A/C and AMP and 100% for OX and P (out of 5 tested isolates in India [49]), 23% for CIP and 50% for CLI (out of 26 tested isolates in Greece [19]), 77.2% for C and 86.3% for TET (out of 22 tested isolates in Iran [66]), 42.86% for ERY, 23.81% for GEN and 9.52% for SXT (out of 21 tested isolates in South Africa [58]) and 13.4% for VAN (out of 14 tested isolates in Turkey [67]). In light of increasing reports of MDR *L. monocytogenes* isolates [58,68], the effective monitoring of the antibiotic resistance in *L. monocytogenes* environmental, food and clinical isolates is very important in order to assess therapeutic options in affected individuals in both veterinary and human medicine settings.

Given the pathogen’s frequency of occurrence and wide distribution in the environment of dairy farms [69,70], the complete exclusion of *L. monocytogenes* from the BTM supply appears to be practically ineluctable. Therefore, the strict adherence to good hygiene practices in bovine farms and in particular during the animal-milking process, the frequent and efficient cleaning of the milking equipment to eliminate biofilms, the rapid and efficient (<4 °C) cooling of the BTM, the rapid transportation of the BTM to the processing facilities and the avoidance of consumption of raw milk or raw-milk dairy products, collectively, should minimize listeriosis health risks for the consumers.

In our study, no data were collected on the total bacterial counts (microbiological quality) of the tested BTM samples. Nonetheless, previous studies revealed no relationship between the total bacterial count and the occurrence of pathogenic bacteria in bovine milk [31]. In addition, recently, Lianou et al. [71] conducted an extensive and comprehensive study on the occurrence of *Listeria* spp. in the BTM of small ruminant farms throughout Greece (325 dairy sheep flocks and 119 goat herds). The authors reported very low isolation frequencies, 0.3% for *L. monocytogenes* (95% CI: 0.1–1.7%) and 0.9% for *L. ivanovii* (95% CI: 0.3–2.7%), and after assessing multiple variables (milk-, climate-, human resources- or husbandry-related variables) that could be potentially associated with the presence of *Listeria* spp. in BTM, no significant effects were noted in terms of milk-related variables such as somatic cell counts, total bacterial counts or gross chemical composition (fat and protein contents) of milk.

In this study, we assessed the occurrence and populations of *L. monocytogenes* in bovine BTM and proceeded to a basic characterization of the isolates. Further detailed genetic characterization of these isolates may shed light into their public health significance, and in light of the recently documented association between hypervirulent *L. monocytogenes* clones and dairy products [72].

## 4. Conclusions

Our study revealed the occurrence, serotypes, virulence determinants, biofilm-forming ability and antimicrobial susceptibility of *L. monocytogenes* in the bovine BTM supply in northern Greece. Considering that *L. monocytogenes* is recognized as a serious threat to public health, the key findings of the current study related to the carriage of virulence genes and the multidrug resistance of the isolates highlight the importance of continued monitoring of the pathogen in farm animals.

## Figures and Tables

**Table 1:** Reported prevalence estimates for *Listeria monocytogenes* in bovine bulk-tank milk in Europe in recent years (2010–2023).

Country	Sampling Year(s)	Prevalence % (No of Samples Positive/Number of Samples Tested)	95% Confidence Interval	Reference
Cantabria, Spain	2017–2019	6.0 (2/34)		[38]
Kosovo	2011–2012	2.7 (6/221)		[30]
Cyprus	2014	0.98 (2/205)	0.04–3.72	[29]
Northern Italy (Lombardy & Emilia-Romagna)	2010–2013	2.22 (131/5897)	1.9–2.6	[28]
North-western Italy (Piedmont)	2009–2011	1.87 (2/107)		[27]
Southern & Central Finland	2011	5.5 (10/183)	3.0–9.8	[31]
Estonia ^1^	2008–2010	18.1 (19/105)		[32]
Latvia ^2^	2008–2010	1.23 (3/244)		[39]

^1^ The animal-species origin of milk was not specified. ^2^ The 244 samples of BTM originated from only four different farms.

**Table 2:** Characteristics of the *Listeria monocytogenes* isolates from bovine bulk-tank milk.

Isolate No.	Molecular Serogroup ^1^	Carriage of Virulence Genes	Antimicrobial Resistance Profile ^2^	Biofilm-Forming Ability
1	IIa	*iap*, *inlA*, *inlC*, *inlJ*, *plcA*, *hlyA*	A/C, CLI, P	Moderate
2	IIa	*iap*, *inlA*, *inlC*, *inlJ*, *plcA*, *hlyA*	A/C, CLI, P, FOX	Weak
3	IIa	*iap*, *inlA*, *inlC*, *inlJ*, *plcA*, *hlyA*, *actA*	CLI, ERY, FOX, P, TET	Moderate
4	IIc	*iap*, *inlA*, *inlC*, *inlJ*, *plcA*, *hlyA*, *actA*	A/C, AMP, CLI, ERY, FOX, P, TET	Weak
5	IVb	*iap*, *inlA*, *inlC*, *inlJ*, *plcA*, *hlyA*, *actA*	C, CIP, CLI, ERY, FOX, P	Weak

^1.^ Profile IIa corresponds to strains of serovars 1/2a and 3a, profile IIb corresponds to strains of serovars 1/2b and 3b, profile IIc corresponds to strains of serovars 1/2c, 3c and 7, and profile IVb corresponds to strains of serovars 4b, 4d and 4e. ^2.^ All isolates were susceptible to G, OX, SXT and VAN.

## Data Availability

Data are contained within the article.
